# Gut Bacterial Communities in HIV-Infected Individuals with Metabolic Syndrome: Effects of the Therapy with Integrase Strand Transfer Inhibitor-Based and Protease Inhibitor-Based Regimens

**DOI:** 10.3390/microorganisms11040951

**Published:** 2023-04-06

**Authors:** Tonatiuh Abimael Baltazar-Díaz, Fernando Amador-Lara, Jaime F. Andrade-Villanueva, Luz Alicia González-Hernández, Rodolfo Ismael Cabrera-Silva, Karina Sánchez-Reyes, Monserrat Álvarez-Zavala, Aldo Valenzuela-Ramírez, Susana Del Toro-Arreola, Miriam Ruth Bueno-Topete

**Affiliations:** 1 Departamento de Biología Molecular y Genómica, Instituto de Investigación en Enfermedades Crónico-Degenerativas, Centro Universitario de Ciencias de la Salud, Universidad de Guadalajara, Guadalajara 44350, Mexico; tonatiuhabd@gmail.com (T.A.B.-D.); susana@cucs.udg.mx (S.D.T.-A.); 2 Unidad de VIH, Hospital Civil de Guadalajara “Fray Antonio Alcalde”, Guadalajara 44350, Mexico; fernando.amador@academicos.udg.mx (F.A.-L.); luceroga08@gmail.com (L.A.G.-H.); aldo.protocolosvih@gmail.com (A.V.-R.); 3 Departamento de Clínicas Médicas, Instituto de Investigación en Inmunodeficiencias y VIH, Centro Universitario de Ciencias de la Salud, Universidad de Guadalajara, Guadalajara 44350, Mexico; cabreraismael2@gmail.com (R.I.C.-S.); karina_reyesqfb@hotmail.com (K.S.-R.); monse_belan@hotmail.com (M.Á.-Z.)

**Keywords:** HIV infection, metabolic syndrome, gut microbiota, gut dysbiosis, antiretroviral therapy, inflammation

## Abstract

Antiretroviral therapies (ART) are strongly associated with weight gain and metabolic syndrome (MetS) development in HIV-infected patients. Few studies have evaluated the association between gut microbiota and integrase strand transfer inhibitor (INSTI)-based and protease inhibitor (PI)-based regimens in HIV-infected patients with MetS. To assess this, fecal samples were obtained from HIV-infected patients treated with different regimens (16 PI + MetS or 30 INSTI + MetS) and 18 healthy controls (HCs). The microbial composition was characterized using 16S rRNA amplicon sequencing. The INSTI-based and PI-based regimens were associated with a significant decrease in α-diversity compared to HCs. The INSTI + MetS group showed the lowest α-diversity between both regimens. A significant increase in the abundance of short-chain fatty acid (SCFA)-producing genera (*Roseburia*, *Dorea*, *Ruminococcus torques*, and *Coprococcus*) was observed in the PI + MetS group, while *Prevotella*, *Fusobacterium*, and *Succinivibrio* were significantly increased in the INSTI + MetS group. Moreover, the Proteobacteria/Firmicutes ratio was overrepresented, and functional pathways related to the biosynthesis of LPS components were increased in the INSTI + MetS group. The gut microbiota of patients receiving INSTIs showed a more pronounced dysbiosis orchestrated by decreased bacterial richness and diversity, with an almost complete absence of SCFA-producing bacteria and alterations in gut microbiota functional pathways. These findings have not been previously observed.

## 1. Introduction

Life expectancy has improved in HIV-infected patients [1]. However, a concern that has arisen in recent years is the weight gain that some patients experience with the use of the contemporary recommended antiretroviral therapy (ART) with integrase strand transfer inhibitors (INSTI)-containing regimens [2,3]. A greater weight gain has been reported when combined with tenofovir alafenamide (TAF) [4].

HIV-infected patients who are overweight or obese have a high prevalence of multimorbidity, including hypertension, gout, diabetes mellitus, dyslipidemia, and chronic kidney disease [5]. Moreover, they have a high prevalence of being metabolically unhealthy and developing metabolic syndrome (MetS) [6].

The mechanisms that lead to weight gain with the use of INSTIs remain poorly understood. A study from dolutegravir-treated SIV-infected macaques showed an increase in profibrotic gene expression and a reduction in beige adipocyte marker gene expression in subcutaneous and visceral adipose tissue. Developing beige adipocytes in white adipose tissue promotes energy expenditure and limits lipid storage [7]. In the same study, human adipose stromal cells (ASCs) were exposed to dolutegravir (DTG), bictegravir (BIC), or raltegravir. DTG and BIC inhibited differentiation in beige ASCs and were associated with elevated pro-adipogenic and pro-lipogenic gene expression [7].

An in vitro study in primary preadipocytes in female mice treated with DTG or BIC found that both drugs inhibited differentiation and altered the thermogenesis of brown fat. DTG and BIC suppressed the expression of UCP1 (an essential protein in thermogenesis) in brown adipocytes, accompanied by a decrease in COX IV and GAPDH, which are key in the electron transport chain and glycolysis. Finally, five-day DTG subcutaneous administration inhibited oxygen consumption and energy expenditure in female mice [8].

Another in vitro study reported a significant decrease in leptin and adiponectin in differentiated adipocytes obtained from the abdominal subcutaneous tissue of HIV-uninfected subjects when exposed to DTG for seven days. Interestingly, subsequent exposure to darunavir (DRV) for seven more days partially reversed the levels of both hormones [9].

In addition, a study of the Women’s Interagency HIV Study (WIHS) cohort compared the changes in plasma metabolomic signatures in patients who switched to an INSTI and experienced >5% weight gain against women who maintained or lost weight. Women who switched to an INSTI and had a >5% weight gain showed changes in mitochondrial energy metabolism and amino acid pathways that reflect insulin resistance. These changes occurred soon after the switch to an INSTI, even before any weight gain occurred [10].

Weight gain associated with INSTIs and TAF usage increases the risk of metabolic syndrome (MetS). In this sense, final results from the ADVANCE study showed that the risk of MetS was significantly higher in the TAF/FTC + DTG arm compared to the TDF/FTC + DTG and TDF/FTC + DRV arms (*p* < 0.001), being greater in women. Fifteen percent of all patients and 20% of women taking TAF/FTC + DTG developed MetS [11].

Moreover, INSTIs have been associated with a 31% increased risk of developing incident diabetes mellitus within six months after ART initiation [12]. On the other hand, it has been found a 2.1 times greater risk of development of MetS for HIV-infected subjects treated with a PI-containing regimen [12]. Older PIs (indinavir, nelfinavir, and ritonavir) are associated with more metabolic disorders leading to insulin resistance, dyslipidemia, and lipodystrophy [13]. These metabolic disturbances caused by PIs are related to mitochondrial toxicity, oxidative stress altering the secretion of adipokines, inhibition of adipocyte differentiation, non-competitive inhibition of GLUT-2/4 transporters, and inflammasome activation [14].

HIV-infected subjects have remarkable changes in the gut microbiota composition, related to damage to gut-associated lymphoid tissue (GALT) and bacterial translocation [15]. We have previously reported alterations in the gut microbiota in HIV-infected subjects with MetS [16], characterized by decreased alpha diversity, increased bacteria associated with inflammation and metabolic endotoxemia, and a significant decrease in SCFA-producing bacteria with an anti-inflammatory role. Another group found similar findings with an increase in the relative abundance of Gammaproteobacteria and a reduction in potentially beneficial genera, such as *Ruminococcus*, *Faecalibacterium*, and *Eubacterium* [17]. Few studies have investigated the changes in the gut microbiota associated with the different ART classes, showing certain differences in beta diversity based on the ART class [18,19,20,21]. However, to our knowledge, no study has evaluated the different microbial communities in the gut microbiota associated with ART and the development of MetS. Thus, we assessed the gut microbiota in HIV-infected patients treated with INSTI-containing regimens versus PI-containing regimens and their association with MetS.

## 2. Materials and Methods

### 2.1. Design and Approval of the Study

This is a cross-sectional study carried out at the HIV unit of the Hospital Civil de Guadalajara, Guadalajara, Jalisco, Mexico. The study followed the Ethical Principles for Medical Research Involving Human Subjects outlined in the Helsinki Declaration in 1975 (as revised in Brazil 2013) and was approved by the Ethics Committee of the mentioned hospital (n.061/19 and n.100/19). Written informed consent was obtained from each participant prior to enrollment.

### 2.2. Study Population

Patients were recruited from January 2019 to February 2021. Adults (18 to 60 years) with HIV infection and MetS, as defined by the 2005 NCEP-ATP III criteria [22], were enrolled. HIV-uninfected subjects without MetS were recruited as healthy controls (HCs). HIV-infected individuals received a protease inhibitor-containing (PI + MetS) regimen or an INSTI-containing regimen (INSTI + MetS) for at least one year with an undetectable HIV viral load for at least six months ago. After dividing them by the ART regimen, the study consisted of 16 PI + MetS patients, 30 INSTI + MetS patients, and 18 HCs.

Exclusion criteria for enrollment were pregnant or lactating women, active AIDS-defining infection, hepatitis B or C virus infection, impaired renal function (glomerular filtration rate < 60 mL/min/1.73 m^2^), chronic pancreatitis, celiac disease, malabsorption syndrome, inflammatory bowel disease, thyroid disorders, and bowel surgery (except appendectomy and cholecystectomy). To increase the homogeneity of the stool sample, exclusion criteria included the use of antibiotics, prebiotics or probiotics, immunosuppressants, corticosteroids, non-steroidal anti-inflammatory drugs, vitamins, minerals, or antioxidants within 30 days prior to stool sample acquisition.

### 2.3. DNA Extraction from Stool Samples and 16S rRNA Sequencing

Fecal samples were collected and immediately stored at −80°C. DNA from the PI + MetS group was extracted according to previously published methodology [16]. Briefly, DNA was extracted from 150 mg of frozen feces with Quick- DNA Faecal/Soil Microbe Miniprep Kit (Zymo Research, Irvine, CA, USA) and quantified with NanoDrop™ 2000 spectrophotometer (Thermo Scientific, Waltham, MA, USA). The raw sequences of gut microbiota of the HC group were from a previously characterized cohort from the same community [23]. 16S metagenomic sequencing library preparation was performed as described previously [16]. V3 and V4 regions from 16S were amplified with Platinum Taq DNA Polymerase High fidelity (Invitrogen, Waltham, MA, USA) using the following primers: F-(5′TCGTCGGCAGCGTCAGATGTGTATAAGAGACAGCCTACGGGNGGCWGCAG-3′), R-(5′GTCTCGTGGGCTCGGAGATGTGTATAAGAGACAGGACTACHVGGGTATCTAATCC-3′). PCR was performed following manufacturer protocol. Product was purified with AMPure XP^®^ (Beckman Coulter, Indianapolis, IN, USA) magnetic beads and was quantified with the Qubit^®^ 3 dsDNA HS kit (Invitrogen, Waltham, MA, USA) according to product indications. Next, index incorporation was achieved with Nextera XT Index Kit v2 Set A (No. Cat. FC-131-2001, Illumina, San Diego, CA, USA) via a second PCR amplification. Finally, amplicons were pooled to equimolar concentrations into a 4 nmol/L solution tube, library denaturing, and MiSeq Sample Loading (kit Miseq Reagent V3 600-cycle, Illumina, San Diego, CA, USA) according to protocol.

### 2.4. Bioinformatic Analysis

Microbiome bioinformatics was performed with QIIME2 version 2021.8 [24]. Based on the quality plot of QIIME2 (Phred quality score > 30), raw sequence data were filtered by denoising with DADA2 via *q2-dada2* [25]. A total of 1,087,004 reads across 64 samples in the three groups were processed. DADA2 was executed for each sequencing run. All other parameters were left at their default settings. After denoising, four sequences (from two samples of the PI group) were removed during quality control. The resulting feature tables were merged, resulting in one “master” feature table containing counts of features per sample, where each feature was a unique 16S rRNA gene amplicon sequence variant (ASV). ASVs identified as mitochondria and chloroplasts were removed from the master feature table. Resultant ASVs were aligned with Multiple Alignment using Fast Fourier Transform [26] (via *q2-alignment*) and used to construct a phylogeny with FastTree2 [27] (via *q2-phylogeny*). For taxonomy assignment, a naïve Bayes classifier (via *q2-feature-classifier*, [28] was trained on the Silva 138 database [29,30].

Alpha diversity metrics [31,32,33,34] were computed with QIIME2. Beta diversity metrics (Weighted and unweighted UniFrac distances) and PCoA were generated with QIIME2 Emperor tool [35,36].

Linear discriminant analysis effect size (LEfSe) was obtained with the Galaxy interface [37,38]. The threshold cutoff value of LDA score was 3.90. Differential abundance analysis of taxa was performed with ANCOM [39].

Phylogenetic Investigation of Communities by Reconstruction of Unobserved States (PICRUSt2) pipeline [40,41,42,43,44] was used to predict the functional pathways of each group according to the MetaCyc Database [45]. The ANCOM method was also employed to analyze PICRUSt2 outputs to determine differentially abundant pathways between groups.

Considering the compositional nature of the data, to calculate Firmicutes/Bacteroidetes, Proteobacteria/Firmicutes, *Prevotella*/*Bacteroides* ratios (supplemental material, Equations (S1)–(S3)) and Spearman rank correlation between taxa and HIV-related variables, the centered log-ratio (clr) transformation was applied to the master feature table through MicrobiomeAnalyst server [46]. No filter steps were performed. Transformed-counts were obtained, and the ratios and Spearman’s correlations were performed.

### 2.5. Statistical Analysis

Data normality was determined using the Shapiro-Wilk test. Then, the Student’s t-test or Mann-Whitney U test was employed, depending on data normality. The Fisher’s exact test was applied to evaluate categorical variables. Alpha diversity metrics among groups were compared using the Kruskal–Wallis test. Beta diversity metrics among groups were compared by performing PERMANOVA tests. Both alpha and beta diversity statistical analyses were corrected with Benjamini–Hochberg (BH) multiple testing through QIIME2 package. All statistical tests were two-sided, and a *p*-value or false discovery rate-adjusted *q*-value of less than 0.05 were considered statistically significant. Data were analyzed using SPSS 25.0, unless otherwise specified. Plots were generated utilizing GraphPad Prism version 8.0.2.

## 3. Results

### 3.1. Demographic and Clinical Characteristics of the Participants

Thirty HIV-positive patients diagnosed with MetS were treated with an INSTI-containing regimen, 16 with a PI-containing regimen, and 18 healthy control subjects were included in the study. From the INSTI + MetS group, 19 received bictegravir (BIC), 10 DTG, and 1 elvitegravir (EVG). In the PI group, 12 received darunavir/cobicistat (DRV/c) and 4 atazanavir/ritonavir (ATV/r). Significant differences in NAFLD prevalence, time since HIV diagnosis, time on ART, and nadir CD4^+^ T cell count were observed between groups. No other significant demographic and clinical characteristics differences were observed (Table 1).

### 3.2. Alpha Diversity

Alpha diversity metrics, which estimate the richness and diversity of bacterial species, such as observed features/ASVs and Shannon indices, were significantly lower in both INSTI + MetS and PI + MetS groups than in HC. Among the ART groups, the indices were considerably lower in the INSTI + MetS compared with the PI + MetS group (*p* < 0.001).

**Figure 1:**
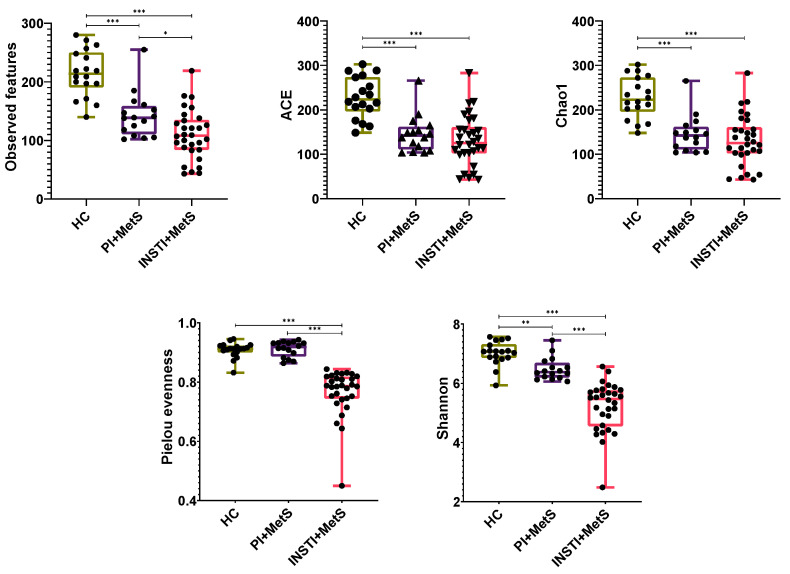
Alpha diversity metrics comparing the HC, PI + MetS, and INSTI + MetS groups. The boxes extend from the 25th to the 75th percentile (interquartile range, IQR), and the lines inside the boxes represent median values. The vertical lines represent the lowest and the highest values. Individual sample values are shown as dots. * *p* < 0.05, ** *p* < 0.01, *** *p* < 0.001.

Likewise, we found a significantly diminished Pielou evenness index in the PI + MetS and INSTI + MetS groups (*p* < 0.001). On the other hand, metrics that weighed underrepresented species (ACE and Chao1) did not show significant differences between the PI + MetS and the INSTI + MetS groups (Figure 1).

As sexual behavior can be a factor that influences the composition of the gut microbiota [47,48], we analyzed the INSTI + MetS and PI + MetS groups according to their sexual behavior. A significant increase in evenness was detected among bisexual (BI) patients, although these only included four patients (Supplemental Figure S1).

### 3.3. Beta Diversity

**Figure 2:**
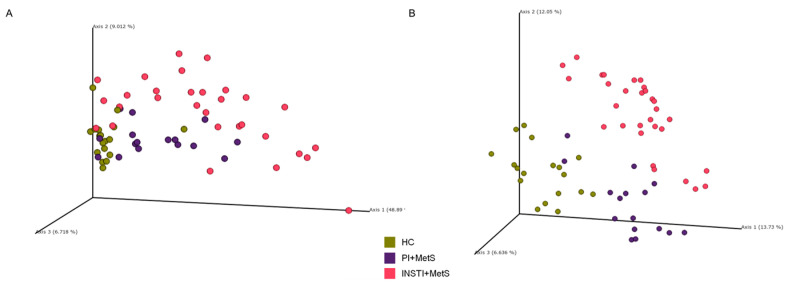
Beta diversity. PCoA plots for weighted (**A**) and unweighted UniFrac (**B**) distances in the HC, PI + MetS, and INSTI + MetS groups.

The principal coordinate analysis (PCoA) for beta diversity distances showed defined groups corresponding to the three study groups, both in metrics that weight quantitative (weighted UniFrac) and qualitative (unweighted UniFrac) aspects (Figure 2). The pairwise PERMANOVA test showed a significant difference among the three groups, verifying a different microbiota profile between them (Table 2). PCoA analysis and PERMANOVA tests were also performed for sexual behavior, as it was done with the alpha diversity metrics. No significant differences were found amongst men who have sex with men (MSM) and heterosexual (HTS) patients (Supplemental Figure S2 and Supplemental Table S1).

### 3.4. Relative Abundances

**Figure 3:**
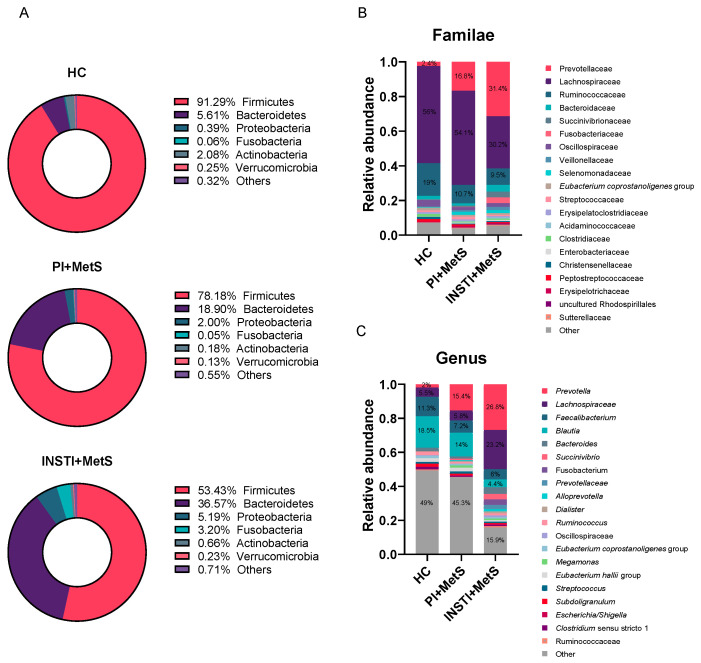
Relative abundances of the plots of the HC, PI + MetS, and INSTI + MetS groups. (**A**) Donut chart representing the relative abundance of different phyla in the three groups. Phyla are accompanied by relative abundance percentage. (**B**) Stacked bar plot of bacterial families in the three groups. Top three families include relative abundance percentage. (**C**) Stacked bar plot of bacterial genera. Top five genus include relative abundance percentage.

**Figure 4:**
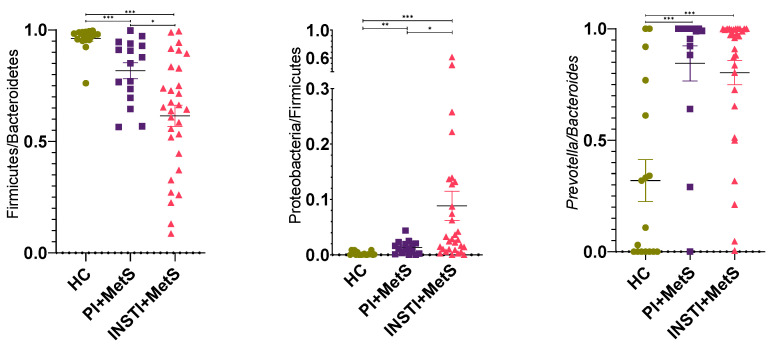
Scatter plot of Firmicutes/Bacteroidetes, Proteobacteria/Firmicutes, and Prevotella/Bacteroides ratios in in HC, PI + MetS, and INSTI + MetS groups. Data were first transformed by means of the centered log-ratio (clr). Then, relative abundances were obtained and calculated according to the material and methods section. Results are showed as mean ± SEM. Analyzed using the Kruskal–Wallis test with Benjamini–Hochberg (BH) multiple testing correction, * *p* <0.05, ** *p* < 0.01, *** *p* < 0.001.

At the phylum level, a decrease in the relative abundance of Firmicutes was observed in the INSTI + MetS group compared to the PI + MetS (53.4% vs 78.2%) and HC (53.4% vs 91.3%) groups. The relative abundance of Bacteroidetes was increased in the INSTI + MetS group compared to the PI + MetS and HC groups (36.6% vs 18.9% and 5.6%, respectively). Furthermore, the relative abundance of Proteobacteria was also increased in the INSTI + MetS group, compared to the PI + MetS and HC groups (5.9% vs 2.0% and 0.39%, respectively) (Figure 3). Following these results, the Firmicutes/Bacteroidetes ratio was significantly decreased in the INSTI + MetS group compared to the PI + MetS and HC groups (*p* < 0.001). On the other hand, the Proteobacteria/Firmicutes ratio was significantly increased in the INSTI + MetS group, compared to the PI + MetS and HC groups (*p* < 0.05) (Figure 4).

At lower taxonomic levels, the genus *Prevotella* had a higher relative abundance in the INSTI + MetS group compared to the PI + MetS group (26.8% vs 15.4%). In contrast, the relative abundance of *Blautia* was higher in the PI + MetS group compared to the INSTI + MetS group (14% vs 4.4%) (Figure 3). The *Prevotella*/*Bacteroides* ratio was found significantly increased in the INSTI + MetS group compared to the PI + MetS and HC groups (*p* < 0.001) (Figure 4). This ratio was also evaluated considering sexual behavior, and no differences were detected (Supplemental Figure S3).

**Figure 5:**
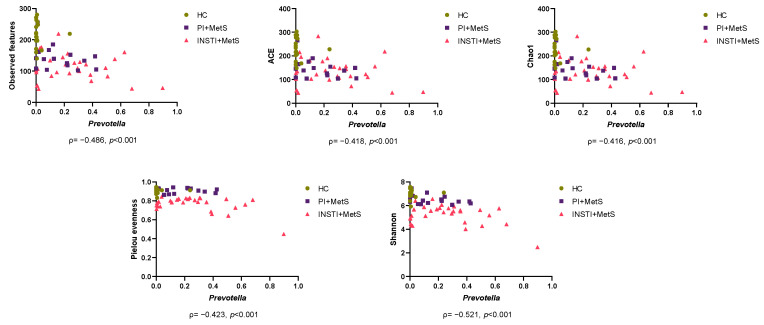
Negative correlations between alpha diversity metrics and relative abundance of Prevotella among the HC, PI + MetS, and INSTI + MetS groups. Spearman’s ρ (rho) and *p*-values (two-tailed) are showed below each diagram. Relative abundances were first transformed using clr as previously described.

As an increase in the relative abundance of *Prevotella* was observed in the INSTI + MetS and PI + MetS groups, we evaluated the correlation between *Prevotella* and alpha diversity indices. The results showed a significant negative correlation between the five assessed alpha diversity metrics (Figure 5).

### 3.5. Linear Discriminant Analysis Effect Size (LEfSe)

**Figure 6:**
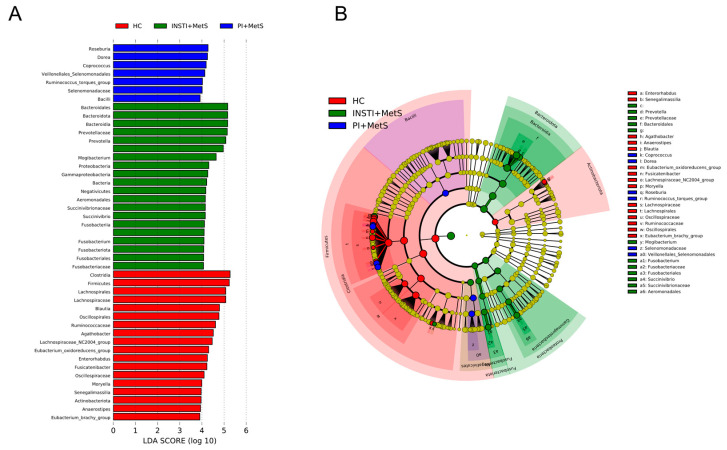
(**A**) LEfSe bar plot showing LDA scores of differentially abundant taxa among the HC (red), INSTI + MetS (green), and PI + MetS (blue) groups (LDA > 3.90, *p* < 0.05). (**B**) Cladogram showing differentially abundant taxa at phylum, class, family, and genus levels between the HC, INSTI + MetS, and PI + MetS groups. Red circles indicate the remarkable taxa in the HC group, while green and blue circles indicate the INSTI + MetS and PI + MetS groups, respectively. (LDA > 3.90, *p* < 0.05).

To identify significant differences in key microorganisms among the three groups, a LEfSe analysis was performed, which revealed a significant increase in the abundance of genera known to be producers of short-chain fatty acid (SCFAs), such as *Roseburia*, *Dorea*, and *Coprococcus* in the PI + MetS group. *Prevotella*, *Fusobacterium*, and *Succinivibrio* genera were significantly more abundant in the INSTI + MetS group (Figure 6).

### 3.6. Differential Abundance: Analysis of Compositions of Microbiomes (ANCOM)

**Figure 7:**
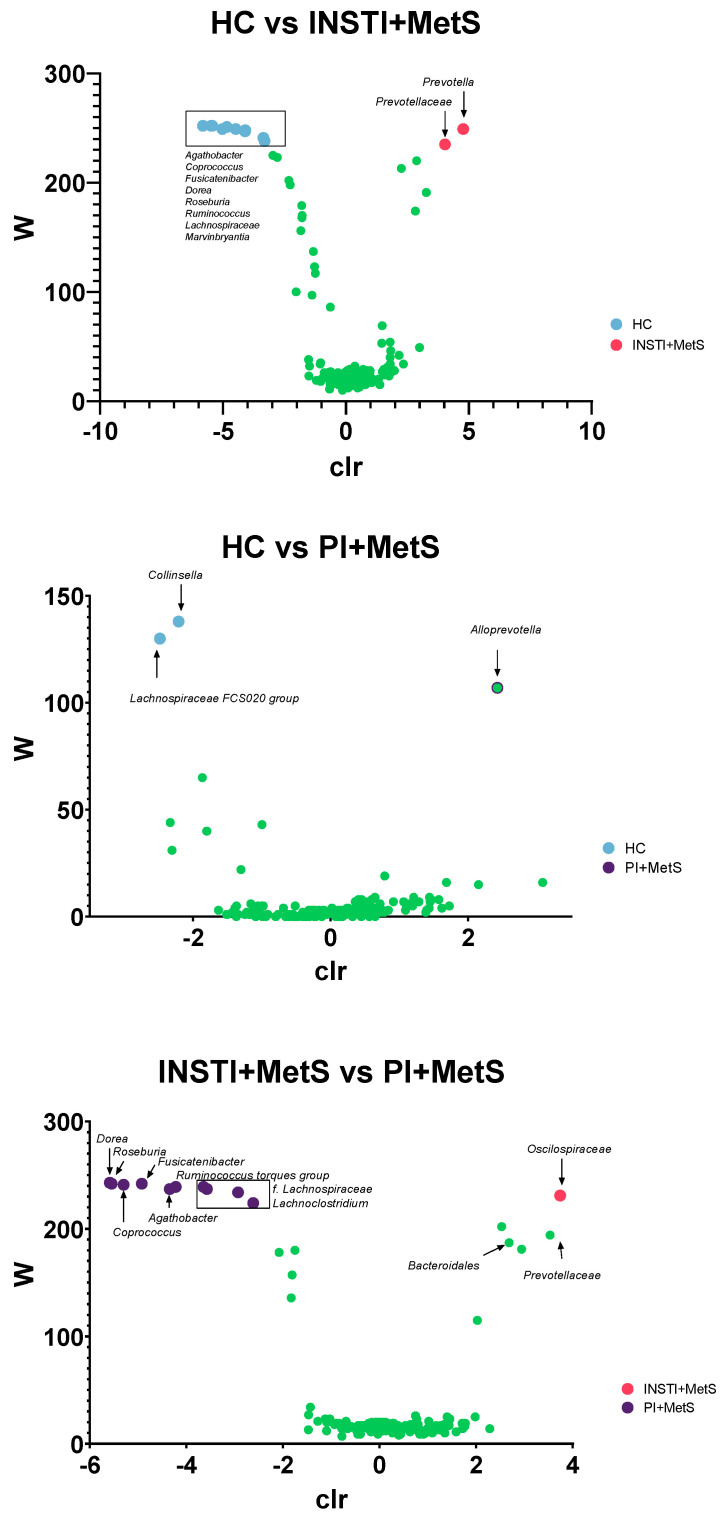
Volcano plot depiction of ANCOM analysis. The horizontal axis is the centered log ratio transformation (clr) representative of the difference in abundance of a significant taxonomical unit. A green color indicates an overlap of ASVs between groups. The W statistic indicates the value of the statistical test corresponding to the number of times the null hypothesis was rejected for each taxon. (Upper) Comparison among the HC vs INSTI + MetS groups, (middle) HC and PI + MetS groups, and (bottom) INSTI + MetS vs PI + MetS groups.

To consider the compositional nature of the microbiome data and strengthen the results, we analyzed the differential abundance between taxa of the different groups using ANCOM, a methodology that does not assume distributions in the population. We found that *Prevotella* genus was strongly associated with the INSTI + MetS group, in comparison to the HC group. Once we analyzed the INSTI + MetS vs PI + MetS groups using this method, we found that *Dorea*, *Roseburia*, *Coprococcus*, *Fusicatenibacter*, *Agathobacter*, *Ruminococcus torques* group, and *Lachnoclostridium* genera were associated with the PI + MetS group. Notably, these genera are anaerobes known to produce SCFAs and other metabolic activities considered beneficial (Figure 7).

### 3.7. Predictive Metagenome Functions

**Figure 8:**
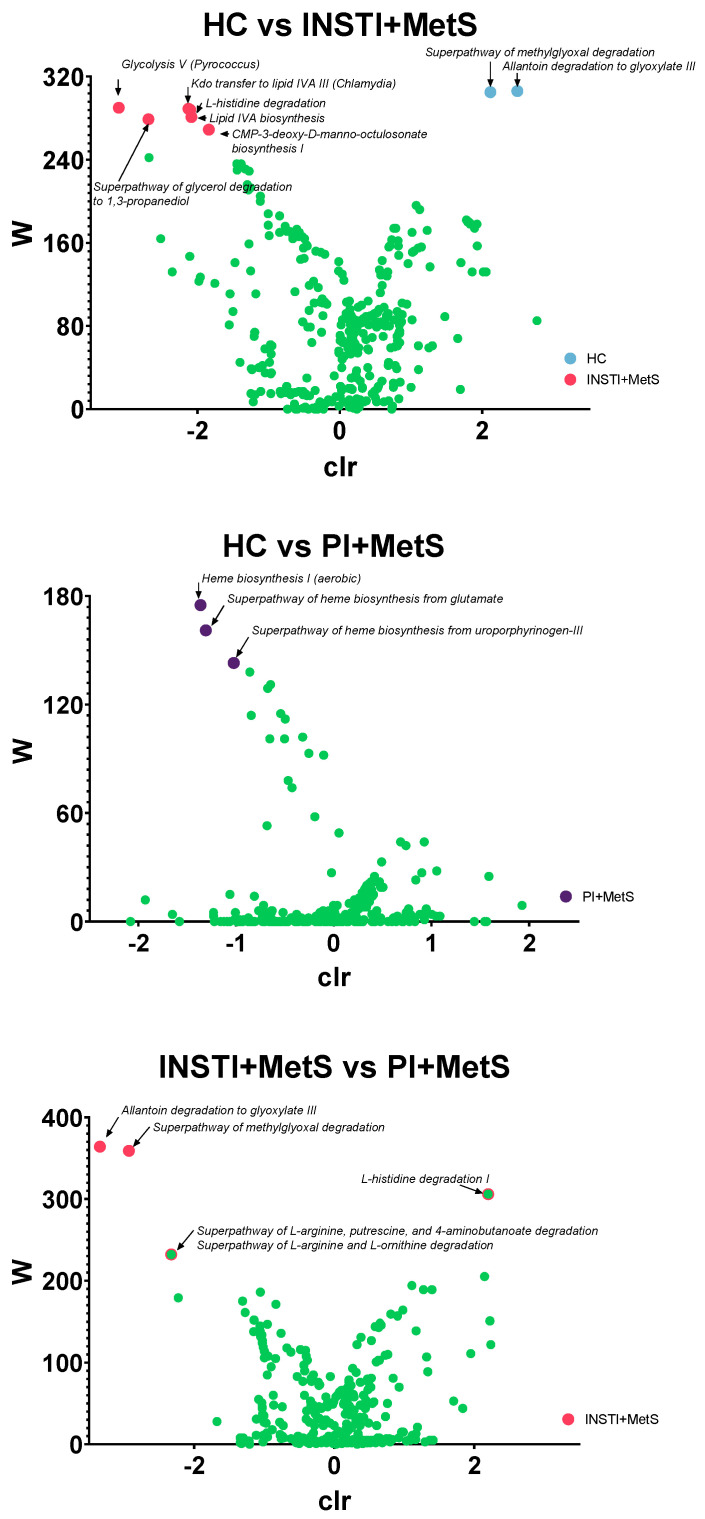
Volcano plot depiction of ANCOM methodology applied to PICRUSt2 results. Each dot represents a statistically significant pathway enriched in each group. Green dots indicate non-significant pathways. Upper: Healthy controls vs the INSTI + MetS groups; middle: Healthy controls vs. the PI + MetS groups; bottom: the INSTI + MetS vs PI + MetS groups.

The ANCOM methodology was applied to the Phylogenetic Investigation of Communities by Reconstruction of Unobserved States 2 (PICRUSt2) results to predict the functional potential of a bacterial community based on marker gene sequencing profiles. We found an increase in functional pathways related to the biosynthesis of LPS components in the INSTI + MetS group (NAGLIPASYN-PWY, PWY-1269). Compared to the HC group, pathways related to the biosynthesis of the heme group were significantly enriched in the PI + MetS group (HEME-BIOSYNTHESIS-II, PWY-5918, PWY0-1415). Interestingly, a comparison of the INSTI + MetS and PI + MetS groups showed an increase in degradation/utilization pathways (PWY-5705, METHGLYUT-PWY) and a trend towards increased degradation of arginine, putrescine, ornithine, histidine, and 4-aminobutanoate in the INSTI + MetS group (HISDEG-PWY, ARGDEG-PWY, ORNARGDEG-PWY) (Figure 8).

### 3.8. Correlation Analysis

**Figure 9:**
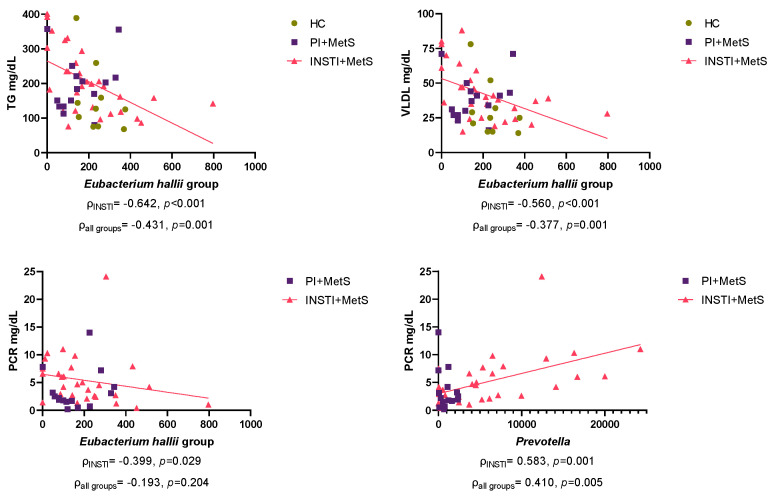
Correlations between blood biochemical parameters and genus of interest in the HC, PI + MetS and INSTI + MetS groups. Spearman’s ρ (rho) and *p*-values (two-tailed) are showed below each diagram. TG: Triglycerides, VLDL: very-low-density lipoprotein. CRP: C-reactive protein. Values on the X-axis are expressed as clr-transformed total abundances.

We further employed Spearman correlations between specific taxa and blood biochemical parameters. Notably, we found significant correlations between *Eubacterium hallii* group and triglycerides (−0.431) and very low-density lipoprotein (−0.377), plus a non-significant correlation between *E*. *hallii* group and CRP levels (−0.193), while *Prevotella* was positively correlated (0.583). Remarkably, these correlations became stronger when analyzing only the INSTI + MetS group (Figure 9).

## 4. Discussion

A remarkable increase in the prevalence of MetS and longevity has been observed in the last decades in HIV-infected subjects with the consequent increase in cardiovascular risk [1,49,50]. A current concern with the use of recommended ART is the increased weight gain in HIV-positive subjects that occurs particularly with the use of INSTIs and TAF [2]. Few studies have explored the impact of ART on gut microbiota and its potential association with weight gain and MetS development [18,19].

We found a decrease in alpha diversity metrics (richness/evenness) in both treatment groups of HIV-positive subjects compared to HC. However, a significantly more pronounced decrease was observed in the INSTI + MetS group, which could suggest a lower recovery of diversity in the INSTI + MetS group. A reduction in alpha diversity has been associated with an increase in the prevalence of MetS in HIV-infected subjects [16,17]. Villoslada-Blanco et al. reported similar alpha diversity (observed features and Chao1 index) in HIV-infected subjects treated with INSTIs compared to healthy HIV-uninfected controls, suggesting a restoration of alpha diversity due to the antiretroviral therapy (ART) [21].

In contrast to our results, Villanueva-Millan et al. found no difference in alpha diversity in the INSTIs group compared to HIV-uninfected controls, and a significantly increased alpha diversity compared to the PI treatment group, after at least one year of treatment [19]. A reason for these differences is that our study only included HIV-infected patients with MetS. We believe that the presence of metabolic alterations in HIV-positive subjects substantially modifies the microbiota profile under the different ART regimens that we validated in this study.

Pinto-Cardoso et al. observed lower alpha diversity in HIV-positive individuals on long-term ART (at least two years) with efavirenz (EFV) and PI (atazanavir/r or lopinavir/r) than healthy HIV-uninfected subjects indicating an incomplete diversity restoration, regardless of the regimen used [18]. It is interesting to observe that Pielou evenness, which is an indicator of the balance in the gut microbiota, did not show differences between PI + MetS patients and uninfected healthy controls, but a significantly lower value was found in the INSTI + MetS group, reflecting an uneven distribution of bacterial taxa in the gut microbiota in the latter group.

The PCoA of beta diversity distances clearly showed defined groups corresponding to the three studied groups in both weighted and unweighted UniFrac distances, indicating strongly that the different clusters could be associated with the ART regimen used. The differences were significant among the three groups. This partially agrees with the results of the previous study by Pinto-Cardoso [18], where they observed differences in beta diversity corresponding to two ART regimens.

We observed a lower relative abundance of Firmicutes with an increased relative abundance of Bacteroidetes in the INSTI + MetS group compared to the PI + MetS and HC groups at the phylum level. The Firmicutes/Bacteroidetes (F/B) ratio was significantly decreased in the INSTI + MetS group compared to the PI + MetS and HC groups. The F/B ratio is widely accepted to have an important influence in maintaining normal gut homeostasis [51]. However, contradictory results have been observed in the F/B ratio related to obesity. While some studies have described an increase in the F/B ratio in obesity [52,53], others have found a F/B ratio decreased in obese HIV-uninfected people [54,55]. Therefore, changes in the F/B ratio in obesity are uncertain and its usefulness in this context is still controversial [56].

Conversely, we observed a significant increase in the Proteobacteria/Firmicutes ratio in the INSTI + MetS group (*p* < 0.001) and in the PI + MetS group (*p* < 0.01), compared to the HC group. Moreover, the INSTI + MetS group significantly increased this ratio compared to the PI + MetS group (*p* < 0.05). Although this ratio has been scarcely reported, a higher number reflects an increase in the relative abundance of Proteobacteria phylum, which encompasses potentially pathogenic and pro-inflammatory species, at the expense of a decrease in species of the Firmicutes phylum. A previous report by Villanueva-Milán showed a significantly higher Proteobacteria/Firmicutes ratio in HIV-positive patients treated with a regimen with INSTIs [19].

We observed an increased *Prevotella*/*Bacteroides* ratio in the INSTI + MetS and PI + MetS groups than in the HC group. Increased abundance of *Prevotella* has been observed in HIV-infected subjects with or without ART [16,21,57]. However, some studies have found that these *Prevotella*-rich, *Bacteroidetes*-poor enterotype could be related to sexual behavior since it has been observed in MSM, regardless of HIV infection [47,48,57].

Considering that sexual behavior could be a major factor affecting the composition of the gut microbiota, we carried out paired analyses of alpha diversity, beta diversity, and taxonomy via ANCOM between the INSTI and PI groups considering this variable. As previously described, only Pielou’s evenness index showed a significant increase among BI patients. Taxonomic analysis considering sexual behavior showed that the *Eubacterium xylanophilum* group was associated with the group of BI patients (Supplemental Figure S4 and Supplemental Table S2); however, the number of patients within this category was low (*n* = 4).

The findings of the significant negative correlation found in our study between the relative abundance of *Prevotella* with the metrics of alpha diversity are in line with the results of a study that found this same negative correlation in HIV-infected subjects, indicating that gut microbiota *Prevotella*-rich correlates with poor microbial diversity [18].

Comprehensively, the LEfSe and ANCOM analyses revealed remarkable findings. *Dorea*, *Roseburia*, *Coprococcus*, *Fusicatenibacter*, *Agathobacter*, *Ruminococcus torques* group, and *Lachnoclostridium* genera were found to be increased in the PI + MetS group. Most of these bacterial genera are important producers of butyrate, which uses the acetyl CoA pathway [58,59,60], except *Lachnoclostridium*, which could synthesize butyrate via 4 aminobutyrate/succinate [61].

These genera are involved in the anaerobic fermentation of non-digestible dietary fibers and resistant starch in the colon, producing SCFAs [62]. They have a known role in maintaining the gut barrier integrity by being a source of energy for colonocytes, increasing the expression of tight-junction proteins, and promoting mucus production and antimicrobial peptides secretion by the intestinal epithelial cells [62,63,64,65]. It has been demonstrated that their anti-inflammatory effects in obese subjects are mediated by reducing the production of TNF-α and IL-6, and upregulating NF-κB related genes in LPS-stimulated adipose tissue macrophages [66].

In addition, SCFAs participate in the metabolism of glucose and lipids through several ways, such as the decrease of hepatic gluconeogenesis, increase in insulin secretion, decrease in glucagon secretion, and fatty acid oxidation [67]. SCFAs improve insulin sensitivity in skeletal muscle through an AMPK-dependent pathway, increasing glucose intake via GLUT-4 [68]. Moreover, it has been shown that propionate increases lipoprotein lipase’s expression and reduces obesity-associated inflammation [69]. Furthermore, SCFAs also reduce plasma total cholesterol, enhancing fecal excretion of bile acids and promoting the hepatic uptake of plasma cholesterol [70]; SCFAs might play a role in the regulation of blood pressure via Olrf78 and FFAR3 receptors [71].

Hence, we consider that it would be relevant to know, in a subsequent study, the plasma levels of SCFAs of the patients in our study to know the possible impact of ART in HIV-infected subjects with MetS.

The analysis of the intestinal microbiota from a co-occurrence perspective describes interesting findings that strengthen our results. Del Chierico F et al., described *Dorea*, *Blautia*, and *Ruminococcus* as markers of pediatric NAFLD [72]. Ottosson et al. associated these three bacterial genera with obesity and predictive plasma metabolites of BMI, including glutamate and BCAAs [73].

On the other hand, *Ruminococcus torques*, which was a characteristic hallmark in the PI + MetS group in our study, is known as a mucus degrader by secreting several different extracellular glycosidases that decrease the gut barrier integrity. It has been associated with irritable bowel syndrome, intestinal bowel disease, and elevated blood triglycerides [74,75,76]. Therefore, undoubtedly, the bacterial profile observed in the PI + MetS group matches some characteristic alterations of the MetS, as well as with the GALT dysfunction associated with HIV infection.

The most outstanding findings found in the INSTI + MetS group at the genera level were an increase in the abundance of *Prevotella*, *Succinivibrio*, and *Fusobacterium*. Notably, *Succinivibrio* was found to be 3-fold more abundant in a HIV-uninfected Mexican obese women group, compared to a normal weight group [77]. Another study found that this genus was overrepresented in an omnivore overweight group vs a vegetarian/lacto-ovo-vegetarian groups, also showing higher values of insulin, HOMA-IR, and a worse lipid profile [78].

*Fusobacterium* was found to be significantly enriched in a group of metabolic unhealthy overweight and obese HIV-uninfected people, in comparison to a metabolically healthy group [79], and in subjects with diabetes mellitus [80,81]. Moreover, the enrichment of this genus in the gut microbiota has been associated with intestinal inflammation [82].

*Prevotella* has been described as an immunogenic commensal with high genetic diversity that may exhibit different properties. Although it has been associated with plant-rich diets and SCFA-producing enterotypes [83,84], some strains have been implicated in having pathobiontic properties linked to cardiometabolic diseases, including obesity, insulin resistance, and MetS [85]. In addition, *Prevotella* and its increased abundance have been associated with other diseases and conditions such as inflammatory bowel disease, rheumatoid arthritis, cirrhosis, NAFLD, insulin resistance, MetS, and low-grade systemic inflammation [23,86,87,88] in HIV-uninfected people. Interestingly, one of these studies in HIV-uninfected Mexican subjects found an increased abundance of *P*. *copri* in patients with MetS and class III obesity. In addition, a positive correlation between serum lipopolysaccharide (LPS) and *P*. *copri* was observed, suggesting this bacterium responsible for metabolic endotoxemia in these subjects [88].

Remarkably, an increased abundance of *Prevotella* has been observed in untreated HIV-infected subjects, associated with colonic T-cell and myeloid dendritic cell activation, inducing pro-inflammatory cytokine production and subsequent systemic T-cell activation [89].

We previously reported an enrichment of *Prevotella* in HIV-infected subjects with MetS compared to an HIV-infected control group without MetS treated with an INSTI-containing regimen [16]. In addition, *Prevotella* was negatively correlated with *Lactobacillus*, *Anaerostipes*, and *Akkermansia*, genera associated with the maintenance of the intestinal barrier, anti-inflammatory effects, and metabolic health [90,91,92].

The absence of representative bacterial genera producing SCFAs in the INSTI + MetS group, associated with the evidenced dysbiosis pointed by the decrease in alpha diversity indices, indicates an important imbalance in the gut microbiota of these patients. This is supported by the fact that although the beta diversity metrics indicate that the three groups conform significantly different profiles, the distance between the microbiota of the INSTI + MetS group compared to HCs is greater than the one between the PI + MetS vs HC groups, as shown by the pseudo-F values.

Added to the above, our findings obtained via the ANCOM methodology do not indicate significant differences regarding these specific bacterial genera between the HC and PI+ MetS groups. Instead, they indicate an upward trend in the genus *Alloprevotella* in the PI + MetS group. This genus has been described as an SCFA-producer genus, and, in some cases, it has been negatively correlated with MetS [93]. However, the similarities of this genus with *Prevotella* are very high [94]. This suggests that the gut microbiota of HIV-infected patients and MetS treated with PIs could be more structurally and taxonomically similar to that of the HC group than to HIV-infected patients treated with INSTIs and MetS.

Our findings related to the enriched functional pathways showed an increase in biosynthesis of LPS components in the INSTI + MetS group, probably associated with the enrichment of Gram-negative bacteria such as Proteobacteria and Bacteroidetes phyla, and the Negativicutes class observed in this group. This correlates with the increase in the Firmicutes-to-Proteobacteria ratio in this group. Negativicutes is an unusual Gram-negative class belonging to the phylum Firmicutes that has been found enriched in the gut microbiota of HIV-infected subjects with CD4^+^ T cell recovery higher than 500 cell/µL. Its abundance was positively correlated with several inflammation markers that mediate an LPS-induced immune response, such as interferon-gamma, IL-1β and chemokines targeting monocytes, including monocyte chemoattractant protein 1 (MCP-1/CCL2, MCP-4/CCL13) and macrophage inflammatory protein 1 alpha (MIP-1α/CCL3) [57]. Concerning LPS functional pathways, it has been found that LPS could induce chronic subclinical inflammation and obesity, leading to insulin resistance [95]. Increased concentrations of plasmatic LPS have been observed in HIV-infected patients, even on ART. Plasma LPS has been shown to have a significant positive correlation with triglycerides and markers of insulin resistance as well [96]. Interestingly, we also found pathways related to arginine degradation in the INSTI + MetS group, which have also been related to the pro-inflammatory state elicited by the LPS-induced IL-6 response (HISDEG-PWY, ARGDEG-PWY, ORNARGDEG-PWY) [97]. These findings encourage follow-up studies to assess the balance of pro-inflammatory/anti-inflammatory cytokines at the systemic level and bacterial translocation markers to corroborate these important predictive analyses.

Correlation analyses showed significant and negative correlations between the *Eubacterium hallii* group and serum concentrations of triglyceride, very low-density lipoprotein, and CRP. These correlations were stronger when only the INSTI + MetS group was analyzed. The *E*. *hallii* group are butyrate-producing commensal bacteria, considered a probiotic [98]. This taxon uses multiple substrates to produce butyrate [99], giving it interesting properties within the intestinal metabolic balance.

In addition, the decrease in its abundance has been associated with childhood obesity in multiple studies [100]. Furthermore, oral administration of *E*. *hallii* has been shown to induce positive effects on metabolism, improve insulin sensitivity, and decrease the adiposity index, presumably through increased butyrate production. Fecal microbiota transplantation from lean donors to MetS patients has significantly increased the abundance of *E*. *hallii*, improved insulin sensitivity, and decreased the adiposity index [101]. Finally, it has been described that the relative abundance of *E*. *hallii* in colonic mucosa has a downward trend in ART-naïve HIV-infected subjects without MetS, compared to seronegative healthy subjects [102]. We suggest that *E*. *hallii* could be considered a bacterial species associated with metabolic health in response to ART.

Our study has limitations that need to be considered. As a single-center study carried out only in the Mexican population, the results cannot be generalized to other geographic regions or ethnic groups. In addition, the diet was not controlled in the populations studied. The IP + MetS group had more time living with HIV infection and less exposure to other ART regimes prior to initiating current treatment. These differences are due to the more recent use of contemporary INSTIs; however, there was no difference in years receiving their current ART regimen between both study groups. Our HC group included only heterosexual participants. However, the HIV-infected patient groups were balanced on this factor. Nevertheless, after controlling for these variables through paired analyses between HIV groups, we did not observe major differences.

## 5. Conclusions

Our study showed a different gut microbiota profile among subjects receiving PI- and INSTI-containing regimens regarding structure, taxonomy, and functions. The group receiving an INSTI-containing regimen showed a more pronounced gut dysbiosis. Periodic analysis of the gut microbiota in the future could contribute to monitoring the unwanted effects of different therapeutic strategies in HIV-infected patients. Further controlled studies in a larger population are needed to clarify the effects of ART therapy on the gut microbiota composition and its contribution to the development of MetS in HIV-infected subjects.

## Figures and Tables

**Table 1:** Demographic and clinical characteristics of participants.

Characteristics	Healthy Control *n* = 18 (%)	PI + MetS *n* = 16 (%)	INSTI + MetS *n* = 30 (%)	*p* Value
Mean age, years (SD)	48.72 ± 8.63	48.75 ± 8.38	42.8 ± 10.1	0.138 ^b^
Gender (men)	11 (61.1)	15 (93.8)	23 (76.6)	0.081 ^d^
Current smoking	0 (0)	8 (50)	9 (30)	0.213 ^‡d^
Current alcohol	0 (0)	10 (62.5)	15 (50)	0.538 ^‡d^
Illicit drug use	0 (0)	5 (31.3)	0 (0)	0.003 ^‡d^
Sexual behavior				
MSM	0 (0)	6 (37.5)	18 (60)	
HTS	18 (100)	6 (37.5)	12 (40)	
BI	0 (0)	4 (25)	0 (0)	
BMI (SD)	25.85 ± 2.86	28.94 ± 4.44	30.5 ± 4.20	0.375 ^‡a^
Diabetes mellitus	0 (0)	3 (18.8)	5 (16.6)	0.580 ^‡d^
Hypertension	0 (0)	5 (31.3)	3 (10)	0.083 ^‡d^
NAFLD	0 (0)	11 (68.8)	5 (16.6)	0.001 ^‡d^
Time since HIV diagnosis, years, median (IQR)	NA	11.50 (5.25–17.0)	6 (2–12)	0.035 ^c^
Time on ART, years, median (IQR)	NA	10.5 (4.25–16.75)	3 (2–10)	0.004 ^c^
Years of current INSTI/PI use, years, median (IQR)	NA	2 (1–2.3)	2 (1–2)	0.700 ^c^
History of ART prior to current INSTI/PI ART	NA	1 (6.3)	16 (53.3)	0.002 ^d^
Current ARV therapy	NA			
Darunavir-containing regimen		12 (75)	NA	
Atazanavir-containing regimen		4 (25)	NA	
Bictegravir-containing regimen		NA	19 (63.3)	
Dolutegravir-containing regimen		NA	10 (33.3)	
Elvitegravir-containing regimen		NA	1 (3.3)	
Absolute CD4+ T Cell count/μL, median (IQR)	NA	740 (331–944)	675 (556–780)	0.800 ^c^
Nadir CD4+ T Cell count/μL, median (IQR)	NA	257 (75–451)	441 (200–628)	0.042 ^c^
HIV-1 RNA	NA			
Undetectable (<50 copies/mL)		15 (93.8)	28 (93.3)	0.130 ^d^
50–199 copies/mL (low-level viremia)		1 (6.3)	2 (6.7)	1.000 ^d^

Abbreviations: IQR = Interquartile range, MSM = Men who have Sex with Men, BI = Bisexual, NAFLD = Non-alcoholic Fatty Liver Disease, SD = Standard deviation, NA = Not applicable. Data normality was determined using the Shapiro-Wilk test. Then, Student’s *t*-test (^a^), Kruskal-–Wallis (^b^), or Mann–Whitney U (^c^) test were employed. Fisher’s exact test (^d^) were applied to evaluate categorical variables. ^‡^ Comparisons within PI + MetS vs INSTI + MetS.

**Table 2:** Pairwise PERMANOVA with BH-FDR tests for both beta diversity distances.

Weighted UniFrac Distance				
**Group 1**	**Group 2**	**Pseudo-F**	***p*-Value**	***q*-Value**
HC	INSTI + MetS	16.586084	0.001	0.0015
HC	PI + MetS	7.825958	0.001	0.0015
INSTI + MetS	PI + MetS	5.420546	0.006	0.0060
**Unweighted UniFrac Distance**				
HC	INSTI + MetS	7.999320	0.001	0.001
HC	PI + MetS	5.323870	0.001	0.001
INSTI + MetS	PI + MetS	5.775082	0.001	0.001

## Data Availability

The data presented in this study are available on request from the corresponding authors.

## Supplementary Materials

The following supporting information can be downloaded at: https://www.mdpi.com/article/10.3390/microorganisms11040951/s1, Supplemental Equations (S1)–(S3); Figure S1: Alpha diversity metrics comparing patient groups according to their sexual behavior; Figure S2: Beta diversity PCoA plots comparing patient groups according to their sexual behavior; Table S1: Pairwise PERMANOVA with BH-FDR tests for both beta diversity metrics distances, according to the sexual behavior of patients; Figure S3: Scatter plot of *Prevotella*-to-*Bacteroides* ratios according to sexual behavior; Figure S4: Volcano plot depiction of ANCOM analysis of differential abundance between taxa according to sexual behavior; Table S2: Percentile abundances of features according to sexual behavior.
