# Supplementary material for: Gut Bacterial Communities in HIV-Infected Individuals with Metabolic Syndrome: Effects of the Therapy with Integrase Strand Transfer Inhibitor-Based and Protease Inhibitor-Based Regimens
Source: Microorganisms. 2023 Apr 6;11(4):951. doi: 10.3390/microorganisms11040951 (PMC10146710; doi:10.3390/microorganisms11040951)
Supplement: Supplementary file 1 [file microorganisms-11-00951-s001.zip › microorganisms-2299906-supplementary.pdf]

Supplemental equations 1 to 3. After centered log-ratio transformation, as described in bioinformatic methods section, the following equations were applied to relative abundance transformed data at phylum (equation 1 and 2) or genus (equation 3) levels:

Equation 1:

$$\boldsymbol{Firmicutes/Bacteroidetes} = \frac{\boldsymbol{Firmicutes}}{\boldsymbol{Bacteroidetes} + \boldsymbol{Firmicutes}}$$

Equation 2:

$$\boldsymbol{Proteobacteria/Firmicutes} = \frac{\boldsymbol{Proteobacteria}}{\boldsymbol{Proteobacteria} + \boldsymbol{Firmicutes}}$$

Equation 3:

$$\boldsymbol{Prevotella/Bacteroides} = \frac{\boldsymbol{Prevotella}}{\boldsymbol{Bacteroides} + \boldsymbol{Prevotella}}$$

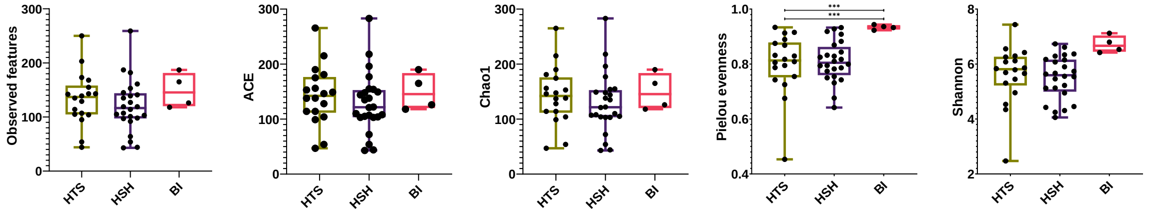

Supplemental Figure S1. Alpha diversity metrics comparing patients groups according their sexual behavior. The boxes extend from the 25th to the 75th percentile (interquartile range, IQR), and the lines inside the boxes represent median values. The vertical lines represent the lowest and the highest values. Individual sample values are shown as dots. HTS: Heterosexual; HSH: Men who have sex with man; BI: Bisexual. * $p < 0.05$, ** $p < 0.01$, *** $p < 0.001$

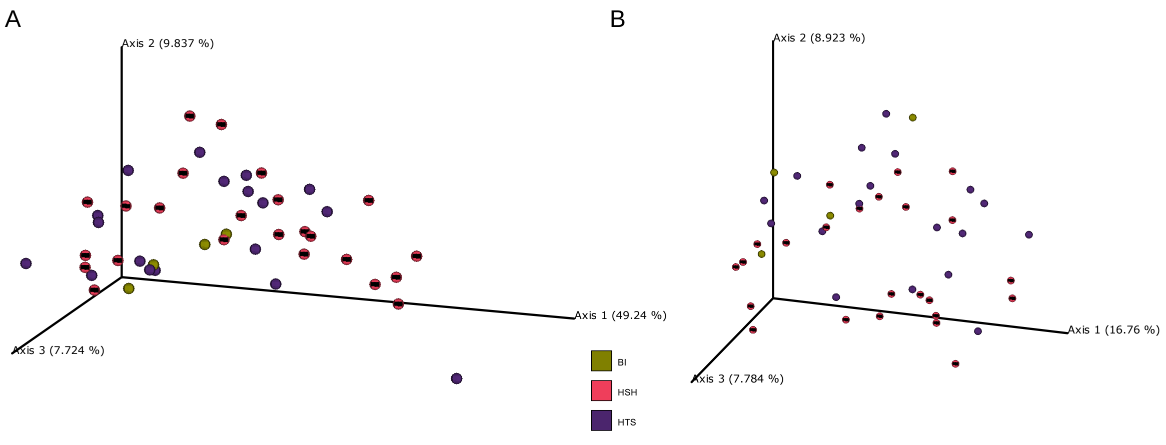

Supplemental Figure S2. Beta diversity PCoA plots comparing patients groups according their sexual behavior. Weighted (**A**) and unweighted UniFrac (**B**) distances. HTS: Heterosexual; HSH: Men who have sex with man; BI: Bisexual.

Supplemental Table S1. Pairwise PERMANOVA with BH-FDR tests for both beta diversity metrics distances, according sexual behavior of patients. HTS: Heterosexual; HSH: Men who have sex with man; BI: Bisexual.

**Weighted UniFrac distance**				
Group 1	Group 2	Pseudo-F	*p*-value	*q*-value
BI	MSM	1.313320	0.222	0.4155
BI	HTS	0.760237	0.550	0.5500
MSM	HTS	1.200126	0.277	0.4155
**Unweighted UniFrac distance**				
Group 1	Group 2	Pseudo-F	*p*-value	*q*-value
BI	MSM	1.815996	0.009	0.027
BI	HTS	1.617057	0.028	0.042
MSM	HTS	1.157605	0.216	0.216

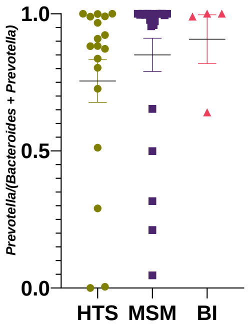

Supplemental Figure S3. Scatter plot of *Prevotella*-to-*Bacteroides* ratios according sexual behavior. Data were first transformed by means of centered log-ratio (clr). Then, relative abundances were obtained and calculated according to material and methods section. Results are showed as mean ± SEM. Analyzed by Kruskal-Wallis test with Benjamini-Hochberg (BH) multiple testing correction. HTS: Heterosexual; HSH: Men who have sex with man; BI: Bisexual. No significant differences were found.

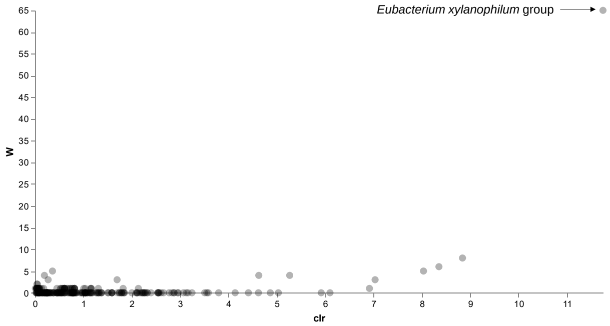

Supplemental Figure S4. Volcano plot depiction of ANCOM analysis of differential abundance between taxa according sexual behavior. The only differential taxon found in the intestinal microbiota of the patients, according to their sexual behavior, was *Eubacterium xylanophilum* group, which is indicated in the graph. This taxon has a higher abundance in the BI group (*n*=4), as described in supplementary table 2.

Supplemental Table S2. Percentile abundances of features according sexual behavior.

Percentile	0	25	50	75	100	0	25	50	75	100	0	25	50	75	100
Group			BI					HSH					HTS		
Eubacterium xylanophilum group	1	27.25	64	139.5	282	1	1	1	1	94	1	1	1	1	119
